# Feasibility of implementing collaborative decision skills training among veterans with psychosis: a qualitative study guided by the practical, robust implementation and sustainability model

**DOI:** 10.3389/frhs.2026.1837307

**Published:** 2026-08-26

**Authors:** Emily B. H. Treichler, Jennisa Bangal, Lauren McBride, Elissa Gomez, Sydney Seaton, Joanna Jain, Han Trieu, Eric Granholm, Gregory A. Light, Borsika A. Rabin

**Affiliations:** 1 Mental Illness Research, Education, and Clinical Center, Jennifer Moreno VA Healthcare System, La Jolla, CA, United States; 2 Department of Psychiatry, University of California, San Diego, La Jolla, CA, United States; 3 Dissemination and Implementation Science Center, Altman Clinical and Translational Research Institute, University of California, San Diego, La Jolla, CA, United States; 4 San Diego State University/University of California, San Diego Joint Doctoral Program in Clinical Psychology, San Diego, CA, United States; 5 San Diego State University/University of California, San Diego Joint Doctoral Program in Public Health, San Diego, CA, United States; 6 Department of Social Work, University of Washington, Seattle, WA, United States; 7 School of Medicine, University of California, San Diego, La Jolla, CA, United States; 8 Graduate School of Psychology, California Lutheran University, Thousand Oaks, CA, United States; 9 Psychology Service, Jennifer Moreno VA Healthcare System, La Jolla, CA, United States; 10 Herbert Wertheim School of Public Health and Human Longevity Science, University of California San Diego, La Jolla, CA, United States

**Keywords:** collaborative decision-making (CDM), implementation science, PRISM, qualitative research, recovery-oriented care, serious mental illness, veterans

## Abstract

**Background:**

Collaborative Decision Skills Training (CDST) is a promising group intervention for adults with serious mental illness (SMI) intended to increase collaborative decision-making (CDM) at the patient-clinician level. CDST previously underwent a systematic, community-engaged mixed methods adaptation to increase fit and feasibility for Veterans with psychosis at VA Psychosocial Rehabilitation and Recovery Centers (PRRCs). This qualitative study applies the Practical, Robust Implementation and Sustainability Model (PRISM) framework to identify the fit and feasibility of the adapted version of CDST within VA PRRCs for Veterans with psychosis.

**Methods:**

Nine Veterans with psychosis and two clinicians who participated in a CDST pilot trial at a VA PRRC provided feedback about CDST in qualitative interviews. PRISM-mapped guides were used to administer semi-structured interviews and develop qualitative codes. Following the initial coding of interviews, four analysts applied a systematic thematic approach to identify themes within each PRISM domain, inclusive of the Reach, Effectiveness, Adoption, Implementation, and Maintenance (RE-AIM) evaluation framework housed within PRISM. Identified themes were then reviewed by three analysts who reached consensus on the final themes to be presented in this paper.

**Results:**

Five cross-cutting themes were identified across PRISM domains, capturing CDST's conceptual alignment with PRRCs and the recovery model; variability of Veteran and clinician interest despite fit or effectiveness; the need for structural integration for sustainability; mixed perceptions of CDST's virtual format; and challenges with at-home practice completion. Additional themes were identified under PRISM Intervention Characteristics, Implementation and Sustainability Infrastructure, Recipients, and all RE-AIM outcomes which inform Veterans' and clinicians' implementation viability and sustainability.

**Conclusions:**

Application of the PRISM framework facilitated identification of multilevel determinants for CDST feasibility. CDST shows promising alignment with the recovery model and the structural approach to care at PRRCs, but delivery mode and auxiliary practice group require adjustment to support CDST's sustainability.

## 1 Introduction

The United States' Veterans Affairs (VA) significantly advanced mental health programming in the past 20 years. One major investment was expanding Veteran-centered care, including recovery-oriented care within Psychosocial Rehabilitation and Recovery Centers (PRRCs) for Veterans with serious mental illnesses (SMI) (1, 2). PRRCs provide holistic, interdisciplinary outpatient services encompassing recovery coaching, medication management, group and individual therapy, supported education and employment, and peer support (2, 3). The recovery lens of these services is intended to imbue care with an empowering and trauma-informed approach that supports Veterans to reach personally meaningful goals even if symptoms continue (4, 5).

While VA's investment led to meaningful innovations in care (e.g., expansion of recovery-oriented care in acute inpatient units) (4, 6), there remain significant gaps in implementation. For example, Veteran engagement and empowerment are essential aspects of recovery-oriented care requiring further investment. Collaborative decision-making (CDM) is a multi-level approach to enhancing patient empowerment in decision-making with benefits including improved treatment engagement and satisfaction; improved personal recovery, quality of life, and social functioning; and reduced symptom severity (7). However, barriers to implementation of CDM exist at patient, clinician, and system levels, including lack of resources, especially for implementing evidence-based practices; and clinician beliefs that people with SMI cannot engage in decision-making (7, 8).

Collaborative Decision Skills Training (CDST) is a promising intervention intended to increase CDM at the patient-clinician level (9, 10). CDST is a solution-focused group intervention that applies evidence-based strategies for adults with SMI to initiate and engage in CDM within a patient empowerment context. CDST was developed and piloted in a civilian setting (9), and recently adapted for Veterans with psychosis participating in VA PRRCs using an iterative, partner-engaged mixed method approach (11). Most adaptations were small in size and scope (12) and intended to improve effectiveness based on the Reach, Effectiveness, Adoption, Implementation, and Maintenance (RE-AIM) evaluation framework (13).

Following adaptation, initial assessment of CDST's feasibility and acceptability within the VA PRRC context and among Veterans with psychosis is needed prior to large scale evaluation (10). Given the multilevel and contextual nature of CDM and CDST, the Practical, Robust Implementation and Sustainability Model (PRISM) is a natural fit for evaluation at this stage (14, 15). PRISM has been effectively used to qualitatively assess implementation factors in VA settings (15) and shows promise in illustrating complex, equity-relevant intersecting contextual and outcome factors through participatory strategies (16). Use of PRISM also allows for continued integration of RE-AIM as in CDST adaptation activities to date, given that PRISM is the contextual expansion of RE-AIM and houses the five evaluation outcomes within it (17).

In this study, PRISM guided our longitudinal qualitative interview data and analysis process during a small open trial of CDST within a VA PRRC serving Veterans with psychosis. Our study goals were to identify Veteran and clinician perspectives on 1) CDM broadly and CDST specifically, especially as it relates to its fit, relevance, and feasibility; and 2) determinants that might influence CDST implementation both within the context of Veterans with psychosis and VA PRRCs.

## 2 Methods and materials

This study was approved by San Diego VA IRB (IRB #H190127). Additional information about this study including the intervention is available in a protocol paper describing the open trial (10). See Table 1 for common acronyms and initialisms included in this paper and Supplementary Data Sheet 1 for PRISM-infused interview guides (18).

**Table 1:** Key for common acronyms and initialisms used.

CDST	Collaborative Decision Skills Training (intervention)
CDM	Collaborative decision-making (practice)
SCALIE	Specify the problem, Consider the possible solutions, Assess your possible solutions, Lay out a plan, Implement, and Evaluate
NOW	Name the issue, identify your Opinion, decide Who you want to be involved in your decision
PRISM	Practical, Robust Implementation and Sustainability Model
PRRC	Psychosocial Rehabilitation and Recovery Centers (setting)
RE-AIM	Reach, Effectiveness, Adoption, Implementation, and Maintenance
VA	Veterans Affairs

### 2.1 Participants

Participants included all nine Veterans who participated in the CDST pilot. These Veterans were currently attending a PRRC for psychosis-related concerns in Southern California and consented for these interviews during their general consenting for the pilot study. Eligibility criteria aimed to maximize representativeness with the PRRC population. Inclusion criteria was: 1) currently receive services in the PRRC; (2) SMI diagnosis (e.g., schizophrenia); (3) age 18 or above; (4) fluent and literate in English; (5) agree to have up to 8 usual care individual mental health appointments audiotaped. Exclusion criteria was: 1) primary substance use or organic neurological disorder diagnosis; (2) determined by PRRC and/or study staff that study participation introduces significant risk to self or others that cannot be reasonably mitigated.

Two VA clinicians who delivered CDST during the pilot were also recruited. These clinicians were eligible due to their participation in the pilot and consented only to the qualitative interviews. The sample together therefore was eleven, which is within the range of estimates for effective qualitative interview samples (i.e., 9–17 people) (19). Further, both systematic review and the tenets of information power suggest that smaller samples are appropriate when study aim is narrow and a specific theory is applied (19, 20); both are true in this case, increasing confidence that the sample will be sufficient.

### 2.2 Study design

#### 2.2.1 Intervention

CDST sessions include 1) agenda setting; 2) at-home practice review; 3) discussion and practice of a coping skill to support confidence using CDM; 4) discussion and practice of one or more specific skills or resources related to CDM (i.e., goal setting, assertiveness, problem-solving, managing conflict), including application to Veteran experiences and goals; 5) session review; and 6) at-home practice assignments. This structure is infused with an empowerment and recovery-oriented lens intended to support Veterans in gaining comfort and confidence to use the knowledge and skills they acquire. Veterans participated in one of two CDST groups, each delivered by one of the clinicians interviewed in this study. Both groups were delivered virtually. Veterans participated in nine 60 min sessions of CDST, offered weekly. There was an optional ∼15–30 min “practice group” offered directly after each CDST session to support Veterans in completing at-home practice assignments. The practice group was led by ET to reduce clinician burden. Clinicians participated in weekly supervision provided by ET. Overall clinical procedures were intended to mirror clinical procedures within the PRRC at the time of the study (e.g., scheduling, delivery, electronic health records).

#### 2.2.2 Qualitative approach

The research team was led by ET, a psychologist and primary developer of CDST, and BR, an implementation scientist and leading expert in PRISM. For all interviews, ET was the primary interviewer due to logistical constraints (e.g., budget, staffing). Most authors are or were VA employees or volunteers during the research period. ET, EG, and GL are long-term VA employees with established relationships in this clinical setting.

Veterans completed interviews at baseline, post-intervention, and three-month follow-up. Veterans could elect to complete an additional interview at post-intervention focused on CDST feedback. Clinicians completed interviews at baseline and post-intervention. The study team aimed to gain and sustain trust with participants through multiple methods including transparency, flexibility, and acceptance of negative feedback. Interviews were semi-structured using interview guides based around the PRISM domains. Interview guides were pilot tested and refined to ensure clarity for participants and alignment with study goals. All interviews were audio-recorded, transcribed by study staff, and quality checked by a second staff member.

The analysis team agreed on a systematic analytic approach using deductive coding of PRISM domains followed by inductive thematic analysis of the content coded within each domain (21–24).

The PRISM-informed interview guides were used to create the initial qualitative codes and code books (i.e., deductive analysis). The initial code books included codes for each of the PRISM domains and RE-AIM outcomes, and then edited and optimized during initial consensus coding to ensure effective coding during analysis. Interview transcriptions were coded by LM, HT, and JJ with supervision and consensus coding by ET and expert consultation by BR using the code books.

Following the initial deductive approach in coding of the transcriptions, four analysts [ET, LM, EG, SS] completed an inductive thematic analysis to identify primary themes associated with quotes coded under each PRISM/RE-AIM domain. The analysts reviewed all of the quotes coded under a given PRISM domain and identified between one and six themes within each domain. Analysts were able to identify themes flexibly, but provide notes regarding decision-making, including quotes that particularly supported identified themes. Effectiveness and adverse effects are not included because they were outside present study scope (i.e., implementation feasibility). Following individual theme identification, each PRISM domain was assigned to two analysts, who first individually reviewed all four analysts' identified themes and proposed a consensus set of themes using a preset consensus worksheet, including combining similar themes and moving themes that appeared to better fit under another PRISM domain. Finally, the two analysts reached consensus on final themes for each domain using a similar worksheet. During this process, the two analysts ranked the top three themes most relevant to this paper, acknowledging that it may not be feasible to present all themes.

After completion of consensus coding, exemplar quotes were identified by JB with supervision by ET. During this process, three analysts [JB, ET, and BR] identified five cross-cutting themes, which represented similar themes that analysts identified in two or more PRISM domains; suggesting these themes had multilevel impact. JB crafted figures to represent these themes and their relationship to each other with supervision and feedback from ET and BR. All authors approved the final analysis and presentation of these results.

## 3 Results

**Figure 1:**
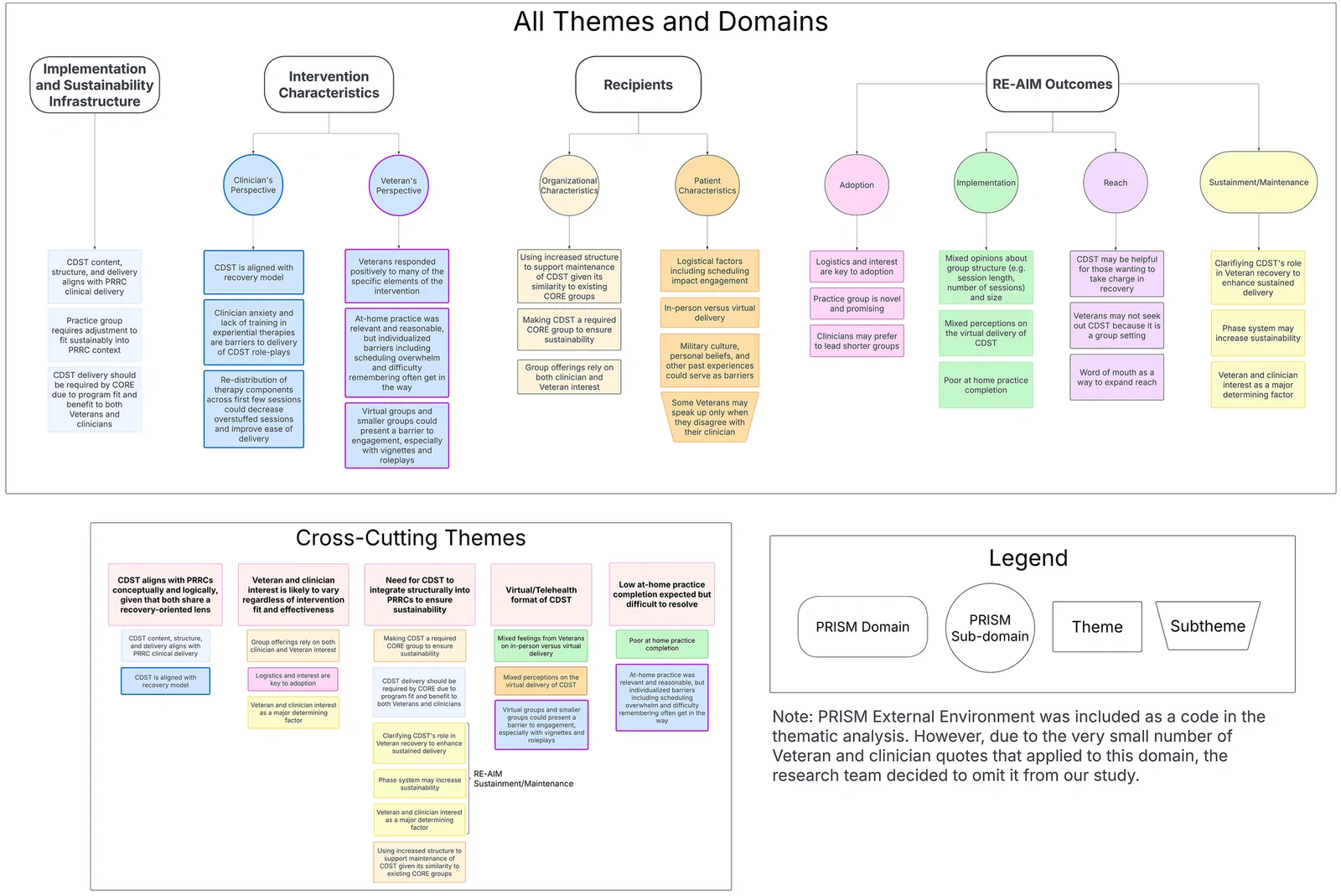
PRISM & RE-AIM domains and themes.

Please see Table 2 for a description of the participants. The three most relevant themes within each PRISM domain are presented here (see Figure 1). For the complete list, please see Supplementary Data Sheet 2. Within these top-ranked themes, we identified five cross-cutting themes that represented at least two PRISM domains (see also Figure 1), presented first below. Additional quotes are presented in Tables 3, 4.

**Table 2:** Participant overview.

Participant	Race and gender	Age range	# interviews completed	# sessions CDST attended
Veteran A	Asian or Asian-American, Female	50–59	3	8
Veteran B	White, Latino, Male	30–39	4	8
Veteran C	White Hispanic or Latino, Male	60–69	3	8
Veteran D	Black/African-American, Male	30–39	4	8
Veteran E	White, Male	30–39	1	8
Veteran F	Black/African-American, Male	40–49	3	8
Veteran G	White, Male	50–59	3	7
Veteran H	White & Native American, American Indian, or Alaskan Native, Male	50–59	4	6
Veteran I	White, Male	30–39	4	3
Clinician A	-	-	2	-
Clinician B	-	-	2	-

Clinician race, gender and age is not reported in this study because it would be immediately identifying in such a limited population.

**Table 3:** Quotes for cross-cutting themes.

Cross-Cutting Theme	Quotes
CDST aligns with PRRCs conceptually and logically, given that both share a recovery-oriented lens	“The group itself fits perfectly. I think it looks, on paper, exactly like every other group that we offer. It's one hour long, it's eight sessions, it's basically a quarter length. It really adheres to all the things in PRRC that we try to have our groups do.” (Clinician B, Pre-intervention).
Veteran and clinician interest is likely to vary regardless of intervention fit and effectiveness	“We offer a wide variety of groups. I think that there is usually a self-selection [bias] in the groups that folks are interested in.. I do think there is a number of Veterans who did not [choose to participate in CDST] and my anticipation is that they would also not [benefit from] the group… [Whereas, the Veterans in CDST] all really want to practice how to assert themselves within their treatment. Some of the [Veterans in CDST] really want to identify what are my goals in treatment and what are my options in treatment. So I'm anticipating it to go really well, just noting the selection bias that we have at PRRC.” (Clinician B, Pre-intervention).
Need for CDST to integrate structurally into PRRCs to ensure sustainability	“How does [CDST] become something that is sustaining power? Or, becomes like a core PRRC group? Some of the groups every year hasn't gotten mentioned again because it's not part of this curriculum that's really a core aspect or something that our PRRC has to run because [all PRRCs] has to run certain units. We have to have a substance use group at least one part of the year. […] I was the only one that signed up for substance use as a supervisor. I'm just saying that reflects like the interests of the staff too so I think.. the interests of staff and trainees that could be a problem.” (Clinician A, Pre-Intervention)
Virtual/Telehealth format of CDST	“I think again [the most challenging part] goes more directly with telehealth and trying to see that everyone was getting all the skills in the manual despite the barriers of telehealth, like they can't “buddy up.” We didn't do as much of any of the interventions within telehealth if that makes sense because they couldn't. They couldn't be like ‘okay let's demonstrate a role-play' and then break into pairs. It was just challenging in that format. So, I don't know if they got the same skills of the intervention as someone would have gotten in face-to-face because of that. That was a concern of mine, but at the same time they seemed like they were attending and got a benefit from it.” (Clinician A, Post-intervention)
Low at-home practice completion expected but difficult to resolve	“I am also looking for a job full-time and I don't have a lot of spare time outside my classes. I had a pretty full schedule at one point where I was taking five classes and the at home practice gets a little frustrating if I have other stuff to do, but I also understand the at home practice is helpful for reinforcing things.” (Veteran H, Post-intervention)

**Table 4:** Additional quotes for Non-cross-cutting themes.

Domain	Themes	Quotes
PRISM Intervention Characteristics – Veteran Perspectives	Veterans responded positively to many of the specific elements of the intervention	“I think [the CDST handbook, the basics card, and the coping skill card] are great and, I want to go back and review it again because I just – even remembering that little part is unusual I normally don’t remember much […] so CDST is helping me. I like the handouts and the pamphlets.” (Veteran G, Post-intervention)
	Logistical factors including scheduling impact engagement	“What was challenging? It was just me trying to figure out my schedule.” (Veteran I, Post-Intervention)
	Military culture, personal beliefs, and other past experiences could serve as barriers	“I don’t really talk to people about what I’ve done, and what I’ve been through. So people really don’t understand where I’m at. I just do their programs even though they don’t know my life experience….And I don’t know if I should tell [therapist at PRRC] about what I’ve gone through. But I don’t have the skills, you know? I always learned on the street. It’s really really hard to say “I have a problem,” you know? “This is a problem,” My beliefs in what I’m doing is [the] Bible. [All] my knowledge [is] in the Bible… my doctor knows about my history but he’s the only one, my psychiatrist, but he’s the only one I’ve ever told. He told me to go see a clergyman, but he wasn’t able to [or] wouldn’t give me too much advice.” (Veteran C, Baseline)
PRISM Intervention Characteristics – Implementers’ Perspectives	Clinician anxiety and lack of training in experiential therapies are barriers to delivery of CDST role-plays	“I noticed that when you try to train others in like experiential therapies there can be a lot of anxiety around some of those activities […] because a lot of people don’t have training in experientials and get quite nervous around them.” (Clinician B, Post-intervention).
	Re-distribution of therapy components across first few sessions could decrease overstuffed sessions and improve ease of delivery	“If you do the NOW model, everybody comes up with an example in session and then treatment team is homework. That might be an easier. That might actually make the session shorter, but also make the homework feel more valuable too, because people really do want to identify who’s on their treatment team versus the NOW model…people weren’t as interested in that I feel like.” (Clinician B, Post-Intervention)
RE-AIM Reach	CDST may be helpful for those wanting to take charge in recovery	Interviewer – “Would you recommend CDST to other Veterans at CORE?” Veteran A – “Yes.” Interviewer – “What would you tell them about why they should participate?” Veteran A – “I would say, it may help you get the results that you want. To me it just seems like a nicer way of requesting something.” [3-Month Follow-up]
	Veterans may not seek out CDST because it is a group setting	Interviewer – “What might make a Veteran less interested or less able to participate in CDST? Veteran B – “If they're uncomfortable with the program or they’re uncomfortable with joining a group.” Interviewer – “What do you think might make them uncomfortable?” Veteran B – “Paranoia and fear… I think that's like the main thing.” [Post-intervention]
	Word of mouth as a way to expand reach	“My best friend now, I told him that I’m attending CDST and he wanted to know how to get into [it]- he’s trying to get help as well, I think. And he asked me about how to ask about it. So, I think word of mouth…I think it can help.” (Veteran G, Post-intervention)
RE-AIM Adoption	Practice group is novel and promising	“It is definitely different from what we’ve done in [the PRRC]. […] This is a new thing and so I think that's where it’s kind of different from the groups that exist.” (Clinician B, Pre-intervention).
	Clinicians may prefer to lead shorter groups	“Are staff less likely to sign up for a ninety-minute group. I guess I never consider that when I sign up for groups. I kind of personally look at the topic, and if it’s a topic that I feel like is beneficial and I am invested in providing that information or that topic to people that's how I pick my group…but I think everybody picks their groups slightly differently. I only get to be here for a year as well, so if I was here longer I might start to value some of those things more, like this is only an hour versus an hour and a half group.” (Clinician B, Post-intervention)
RE-AIM Implementation & Sustainability Infrastructure	Practice group requires adjustment to fit sustainably into PRRC context	“I want it to be about patient choice too and I want them to be empowered to say, ‘Hey, I would like to come to practice.’ But at the same time if it was this idea that the group was an hour and a half and the last half hour is practice, then maybe they would end up doing some of the homework in that practice half hour […] I mean sometimes I felt like towards the end where we were getting some momentum and they were doing the skills and if [the practice group] was mandatory maybe people would have stayed … And then [the skills] would’ve stuck more.” (Clinician A, Post-intervention).
RE-AIM Implementation	Mixed opinions about group structure (e.g. session length, number of sessions) and size	“I thought it could be longer and I thought we could take a break. That way I don’t have to excuse myself during the group.” (Veteran C, Post-intervention).

*Cross-cutting theme #1: CDST aligns with PRRCs conceptually and logistically, given that both share a recovery-oriented lens. [Implementation and Sustainability Infrastructure & Intervention Characteristics: Clinician Perspectives]*

A clear take-home was CDST's overall fit with the PRRC. One reason was that both are aligned with the recovery model, increasing perceived benefits for Veterans. “Learning about how to be more interactive in my providers and recovery, that was very helpful. It gave me a little boost of confidence.” (Veteran H, Post-intervention) Both Veterans and clinicians felt that the content, structure, and delivery of CDST also aligned with PRRC clinical delivery. As clinicians learned more about CDST through training and delivery, the stronger they felt about CDST:

“Knowing more about what [CDST] was, I see the benefit in how it could help some of our patients be assertive, or empower them, I think it's very in line with the recovery model. And knowing that, I think it's more likely that I would want to do it again and maybe that would be the same for other clinicians.” (Clinician A, Post-intervention).

*Cross-cutting theme #2: Veteran and clinician interest is likely to vary regardless of intervention fit and effectiveness. [Organizational Characteristics, Adoption, & Sustainment/Maintenance]*

Although CDST is a natural fit for PRRC, voluntary group offerings rely on both clinician and Veteran interest. Group interventions require a minimum number of Veteran participants for delivery. In this PRRC, groups are offered as often as every 3–4 months, but reliably enrolling enough Veterans to offer the same group this frequently may be difficult:

“This is not CDST specific… but I think one of the big issues that we have with our groups at [our PRRC] is that we don’t necessarily have enough interest [for] each group every quarter. There’s some groups that are really popular. We struggle to get the enrollment we want. We have three or four Veterans who are really passionate about it, but technically we’re supposed to be at six in order to run that group. I think that’s the feasibility thing that would need to be considered if [CDST] moved forward.” (Clinician B, Pre-intervention).

Additionally, clinician interest waxes and wanes even with effective, values-aligned groups, making sustained delivery hard, especially if one specific clinician is expected to deliver a group on an ongoing basis. This is partially due to the PRRC's integrated training programs:

“Our trainees come in, and they have different interests. Like the music mindfulness group a couple of years ago, it seemed like Veterans really enjoyed that group and really were participating in it. But [the trainee providing the group] left. It's fair because it's kind of dependent on the supervisors remembering that model and bringing it [up] that could be of interest. But that group hasn't been run again.” (Clinician A, Pre-intervention).

*Cross-cutting theme #3: need for CDST to integrate structurally into PRRCs to ensure sustainability [Implementation and Sustainability Infrastructure. Organizational Characteristics & Sustainment/Maintenance]*

Despite conceptual and logistical fit (cross-cutting theme #1), broader and more systemic integration is necessary, especially given the characteristics of the setting. Using increased standardization and structure to support maintenance of CDST given its similarity to existing PRRC groups might facilitate long-term delivery*.* Given that this PRRC is one of many PRRCs nationally, clinicians described it within that national context, including clinical and logistical mandates that might influence CDST delivery. For example, PRRCs have required national clinical offerings, and this PRRC, like many, also standardizes some elements of care. Standardization could resolve a number of the potential organizational issues that might prevent sustainability despite CDST's fit for PRRCs:

Interviewer – “What kind of role do you think clinicians might have in CDST's success both in terms of it continuing at [the PRRC] and in terms of it benefitting Veterans?”

Clinician A – “I think if it was a PRRC requirement that they do [like] Illness Management and Recovery] … Some of them [the requirements], like Illness Management and Recovery, is supposed to be one of the first classes Veterans take. I think putting [CDST] in the beginning might be good because there's a lot of stuff like “who's on your treatment team?” and maybe that would be really helpful when Veterans are starting out in our PRRC just to figure out “what a recovery coach is, and who my psychiatrist is, who do I want to be on my treatment team?”” [Post-intervention]

Requiring CDST would also benefit both Veterans and clinicians by facilitating greater CDM into PRRCs:

“If this is another required group, I think that would really help with making sure that it's like a part of our [clinicians'] baseline knowledge [about CDM]. And also maybe providing - as we onboard -some discussion about the decision making process that we go through in recovery planning, and how the aim of that is collaborative decision making.” (Clinician B, Post-intervention)

A clear example of integrating CDST structurally is that many VA PRRCs use a phase system, where Veterans move through phases associated with specific interventions as they progress towards graduation (the last phase). Given that many of the participants in this study suggested CDST would be a helpful introductory group, clinicians suggested that including CDST as a “phase 1” group system may increase sustainability:

“We have a phase system described in the policies and procedures manual, but I don't think it really reflects what we do […] If it was a more structured system and if CDST was thought to be part of one of those phases then it would make sense. [CDST] might stay around longer.” (Clinician A, Post-intervention)

*Cross-cutting theme #4: the virtual/telehealth format of CDST provided both benefits and challenges. [Implementation; Recipients: Patient Characteristics; & Intervention Characteristics: Veteran Perspectives]*

CDST was delivered virtually due to the VA's COVID-19 protections. This format was received ambivalently. One benefit of virtual delivery was increased feasibility of participation, especially for Veterans who lived outside of an easy commuting distance of the clinic or had other conflicts making it difficult to drive weekly to the clinic:

“I thought [telehealth] was very very good. I find it more difficult being in person because I live far away from [city]. I would have to take a shuttle and the shuttle…leaves by 12 o'clock or 1 o'clock [so] the group would have to be in the morning for me.” (Veteran C, Post-Intervention)

However, virtual groups introduced difficulties with engagement among Veterans, especially with interactive components like vignettes and roleplays. This problem was compounded by CDST's group size (4–5 people per group):

“If you have a small group and then one person is super introverted, that makes it difficult. But if you have a larger group and there is one introverted person, then the rest of the group kind of picks up that slack. If [CDST] was in person, the role-playing might be a little bit easier because you'd be in a room with somebody and you'd get a little more comfortable with them.” (Veteran H, Post-intervention).

Difficulties with engagement were especially compounded when Veterans experienced technical problems:

Interviewer – “What didn't you like about CDST?”

Veteran D – “Nothing I wouldn't say I didn't like, but internet connection was slow sometimes. I had to use my phone and I couldn't [always] see the video, see the group, see everyone.” [Post-intervention]

Finally, some Veterans emphasized the benefits of in-person delivery in terms of social connection and engagement for otherwise isolated Veterans with mental illness:

“In-person, it helps also because then you have to get up and go do something. For me, that's good because maybe I'm feeling depressed or something that day so but I have to go to the meeting [but] if I have to do it online then I can just skip it, you know? But it's harder to skip it if you have to go in-person and be there.” (Veteran G, Post-intervention)

*Cross-cutting theme #5: low at-home practice completion was expected but difficult to resolve. [Implementation & Intervention Characteristics: Veteran Perspectives]*

Clinicians observed poor at-home practice completion. Notably, this was the issue that both clinicians predicted in their baseline interviews because they found low completion to be generalized problem without clear cause or resolution.

Interviewer – “Do you think that's a solvable roadblock [at home practice completion]? Can you get it to 100% and how do you do that? How do you accomplish that if you could?”

Clinician B – “You know that is like the million dollar question, Emily. If I had the answer to that I feel like I would be a therapy millionaire.” [Post-intervention]

Veterans mostly described at-home practice as relevant and feasible to complete. Veterans who struggled with completion reported a range of individualized barriers including Veterans' other responsibilities, feeling overwhelmed by sheer number of responsibilities, increases in mental health symptoms, and memory issues.

“I thought [the assignments] were all good and they were very self-explanatory, but I haven't been going over them as of yet either, it's just that it was too much for me at the time.” (Veteran C, Post-intervention)

These barriers were sometimes compounded by ambivalence about perceived value of at-home practice activities.

“I would say most of the at-home practice could have been done while there was a lull [in class] […] I didn't really think that [the homework] added much value to the class itself. They seemed kind of minor.” (Veteran H, Post-intervention)

We describe the remaining top-ranked themes not included within a cross-cutting theme within each PRISM domain below.

### 3.1 PRISM intervention characteristics

#### 3.1.1 Veteran perspectives

Veterans described a range of diverse perspectives to CDST. Veterans responded positively to many of the specific elements of the intervention, including the CDM skills and coping skills, and the physical materials provided:

“It's hard to [talk to a doctor] sometimes when you're in that system … For Veterans to be able to talk with a doctor about what kind of treatment options they want is a really good thing, so I don't think I would have changed anything about [the group content].” (Veteran I, Post-intervention)

“Cause I get angry all the time, I get frustrated when things aren't going my way, so I like the SCALIE model. Most of the time, what people tell me sometimes, I'm not specific, y'know I'm not specifying what the issue is, so I guess I gotta start [like the first step of the SCALIE model].” (Veteran F, Post-intervention)

#### 3.1.2 Implementers' perspectives

Although CDST is aligned with the recovery model and a good fit overall for PRRC (cross-cutting theme #1), one anticipated difficulty was clinician anxiety or lack of training. While the clinicians in this study reported self-efficacy delivering role-plays during the intervention, they predicted potential wariness about role-plays among clinicians who were not already trained in experiential or skills-based therapies. Structurally, clinicians recommended re-distributing materials across the first few sessions to improve ease of delivery*.* The clinicians noted that there are many non-manualized tasks to complete, especially in the initial sessions, to ensure that Veterans are successful in CDST, and having enough time to develop rapport and trust is key to that success:

“If they don't trust you to tell you [when] they're having a conflict with their psychiatrist, they won't engage with the material as well. So, I think that first session is really important for building rapport, all of those introductions, justifying that you believe that Veterans should take an active role in their treatment and you want to support them in that. It just takes a little bit of time to establish that trust especially if it's your first time meeting folks. So maybe taking out some of that content in session 1, and allowing for more relationship building there would be beneficial.” (Clinician B, Post-intervention).

### 3.2 Recipient characteristics

#### 3.2.1 Patient characteristics

Veterans' military background, personal beliefs and other past experiences were all important characteristics to consider:

“I also think it's particularly hard for some of our Veterans and I think a large part of that is [that] what is being assertive in military culture is often what civilians would call aggressive. It can be really hard to modulate for your audience based on your prior experiences.” (Clinician B, Pre-intervention).

Veterans also noted that while cognitive and logistical factors including scheduling impact engagement in CDST, they liked the structure overall:

“If I don't have it on my phone or if I miss putting it on my phone- which I happen to do often -I forget. And one day I look at it and it doesn't have anything on my calendar. But I didn't know about [the group] and I forgot. But I like [CDST] because it makes me, still makes you like accountable for something … to be on time and be there every week.” (Veteran G, Post-intervention)

#### 3.2.2 Organizational characteristics

All of the Organizational Characteristics themes fit well under cross-cutting themes #1–3.

#### 3.2.3 External environment

There were no codes associated with this PRISM domain and therefore no themes identified during the analysis process.

#### 3.2.4 RE-AIM: reach

Veterans and clinicians felt that while CDST was likely to benefit most Veterans, CDST may be especially helpful for those wanting to take charge in recovery*.* Veterans took advantage of that to maintain engagement:

“I thought [the roleplays] were great. I thought that they definitely helped me because even that one week I was depressed, I still came back and most of the time I would not have come back I would have just stayed where I was… I like the role-plays and everything enough and that helped me for my mind to try to be accountable.” (Veteran G, Post-intervention)

However, there may be some Veterans who find groups like CDST undesirable due to social anxiety or other reasons. These issues suggested that limits on Reach may be due to a lack of fit between CDST and some Veterans: “It's hard to operationalize it because I think that some people still have a long way to go in terms of being able to do the skills, but some people start off with skills already, and the ability to do some collaboration.” (Clinician A, Post-intervention).

#### 3.2.5 RE-AIM: adoption

Promise for adoption appeared strong overall. Clinicians felt that there were elements of CDST, especially the practice group, that were novel and promising:

“I'm actually kind of hopeful about the half hour homework group. I'm really curious to see if that model works and I think it could be something, if it does work, that we actually implement for other groups as well. Our Veterans are very good at coming to group if they have the time blocked out.” (Clinician B, Pre-intervention)

At the same time, the at-home practice group added 30 min onto the regular 60 min CDST session. Clinicians might prefer shorter groups and need to weigh that time when deciding whether to offer the group outside of a research context, creating a potential barrier to adoption: “If I was here longer [beyond a training year] I might start to value… like, “this is only an hour versus an hour and a half group” or like, “this group is a lot of admin”– I might start to really think about those things more.” (Clinician B, Post-intervention).

#### 3.2.6 Implementation & sustainability infrastructure

As described above in cross-cutting theme #1, Veterans and clinicians felt that CDST aligned with PRRC in terms of content, structure, and delivery. The exception to this was the practice group, which was new to the clinic and unlike any current offerings. As a result, the practice group requires adjustment to fit sustainably into PRRC context. One Veteran suggested instituting a one-on-one check-in process:

“If you can't do [the practice group] right away after the group, you know – “can we call you back later?” or could you check up on us during the week to see where we're at and that way we'll have our homework done for the next group instead […] maybe after [the group] it should be mandatory for everybody to check in with your doctor, or the facilitator to check in with the students.” (Veteran C, Post-intervention).

However, this might not align with the PRRC's recovery model: “I want it to be about patient choice too and I want them to be empowered to say, “Hey, I would like to come to practice.”” (Clinician A, Post-intervention).

#### 3.2.7 RE-AIM: implementation

Implementation themes were mostly contained in the cross-cutting themes. The remaining theme identified mixed opinions about group structure (e.g., session length, number of sessions) and size among both Veterans and clinicians, who tended to agree that while the content of the group was strong, the sessions were overstuffed, requiring more time to implement feasibly:

Interviewer – “There were 9 sessions and they were each 60 min long. Did that seem right to you? Or would you have preferred it be longer or shorter?”

Veteran B – “I think it's kinda light. I would prefer it if it were a little bit longer so we get more practice.. Yeah, more sessions.” [Post-intervention]

“A thought that I've had about the group that would really be a big change could be [lengthening it]… Race-Based Stress & Trauma is an hour and half and it works within all of our PRRC processes. People really liked that it was an hour and a half because they said that the pace felt slower, and they felt like they got to know each other better than other PRRC groups which are only an hour. Are staff less likely to sign up for a ninety-minute group? I don't know– I never consider that when I sign up for groups… I personally look at the topic and if it's a topic that I feel like is beneficial and I feel invested in providing that information to people like that's how I pick my group.” (Clinician B, Post-intervention)

#### 3.2.8 RE-AIM: maintenance

Maintenance factors were captured in cross-cutting themes #2–3. Additionally, identifying the value- and goal-based alignments between CDST and the clinic were one clear factor, especially in terms of *clarifying CDST's role in Veteran recovery to enhance sustained delivery:*

“What can CDST do to promote Veterans graduating from [the PRRC] or advance their recovery? What's their connection between CDST and them being better able to sustain their recovery after [graduating from the PRRC] and not need to be re-admitted or re-referred? […] I think that there are things that [CDST] teaches, like choice and how to select your treatment team, maybe how to select what organizations you want to be a part of, and that could be very helpful in recovery planning, as opposed to just the clinician telling you what to do. So, maybe that would help [with] how long the Veterans need to be in [the PRRC].” (Clinician A, Post-intervention)

## 4 Discussion

We identified that CDST is conceptually aligned with the recovery-oriented purpose of this clinic and PRRCs nationwide, demonstrating high levels of fit at the clinic-, clinician-, and Veteran-levels. CDST also demonstrated structural fit in many ways (e.g., length of sessions, number of sessions, group modality). These results indicate that the core functions of CDST translated well in the clinical setting, resulting in investment among targeted clinicians and Veterans. At the same time, CDST faces similar barriers that other PRRC interventions experience; most predominantly, that even well-liked, effective interventions are rarely sustained without structural requirements. While some specific elements of CDST likely require adjustment to increase feasibility, the most important determinant for long term success in PRRCs is organizational and structural investment.

Based on these results, it is not a surprise that organizational characteristics appear to strongly influence maintenance. Literature in other areas suggests that proactive leadership; sustainable infrastructure including earmarked funding and contracts; integration of evidence-based practices into a given setting and technical assistance for service providers can increase sustainability of new evidence-based practices in clinical settings (25–27). This study identified that while enthusiasm and buy-in from a given clinician for an intervention like CDST is important, it is not sufficient to maintain intervention delivery given potential changes in staff due to turnover, short-term trainees, and competing service delivery priorities.

One promising avenue to sustainability was via CDST's conceptual fit. Clinicians and Veterans were enthusiastic about the CDM model, and suggested specific implementation strategies to build CDM and CDST into the PRRC's standard of care not only to sustain CDST but to improve overall care quality. These suggestions usually fell under the “change infrastructure” category in the Expert Recommendations for Implementing Change (ERIC) compilation (28, 29). For example, one strategy proposed was integrating CDST as a required “introductory” intervention to the PRRC itself, which might improve overall benefit for Veterans while enhancing clinician knowledge and use of CDM. A prior study evaluating Illness Management and Recovery in VA similarly identified that implementation was more likely in locations with a mandate to implement and/or “sensitive period,” like the development of new, evidence-based program curriculum (30). This suggests that taking advantage of VA's directives to embed recovery-oriented, evidence-based and Veteran-centered care could facilitate structural changes to implement CDM generally and CDST specifically.

The translation to telehealth due to the pandemic was unanticipated (31, 32). There is notable evidence that telehealth is non-inferior in general for delivery of mental interventions (33). Telehealth can increase attendance among people with SMI (34). Our analysis suggests that while telehealth demonstrates clear benefits (e.g., accessibility, increased engagement and attendance), in-person care may be preferred and enhance benefits for Veterans experiencing social isolation. Virtual skills training groups are complex logistically; simultaneously, it is a realistic setting for role-plays to learn CDM given that these Veterans predominately saw their other clinicians virtually. Higher adopters of telemental health within VA tend to perceive video meetings as compatible for their Veteran populations and equivalent in quality to in-person sessions (35). Future research should elucidate best-practice adaptation strategies when moving interventions to telehealth, and identify potential decision points where interventions cannot be feasibly adapted without function loss (36).

### 4.1 Implications and future directions

The qualitative data presented here was uniquely positioned to assess implementation drivers in this clinical context. The approach to embed PRISM throughout data collection and analysis was relatively novel because it is the first to our knowledge to report cross-cutting themes using the PRISM framework, and we utilized this framework at every stage – design, implementation, and analysis. Further, it yielded meaningful findings for a relatively understudied group in implementation science [i.e., psychosis and serious mental illness, cf (37).]. For example, it allowed for nuanced assessment of contextual factors and the integration of themes into cross-cutting themes during the iterative analytic process, which our quantitative data was not able to provide due to differential strengths in data types and methods. The cross-cutting themes themselves—themes which translated across two or more PRISM domains—demonstrate the interconnectedness of these multilevel contextual domains and their impact on everyday clinical processes. These findings illustrate how even relatively small innovations (e.g., our 30 min at-home practice group pilot) are influenced by multilevel implementation determinants. Further, it shows that multilevel context can be meaningfully assessed to support future research and implementation efforts in small implementation pilots like this one. Notably, we did not identify any themes related to the PRISM domain External Environment. This may be because the VA is by nature a closed system (e.g., little contact with insurers). However, future studies which include VA decision-makers, including clinic and hospital directors, heads of national offices, and policy makers, may be more likely to identify themes in this domain.

We will apply these findings by making targeted adaptations prior to hybrid type 1 trial of CDST (e.g., removing the at-home practice group, lengthening by two sessions) (38). Further, we will use this analytic method in the hybrid type 1 trial, especially to assess promising implementation strategies to integrate CDST into existing infrastructure. We recommend that other studies applying qualitative methods 1) utilize potential pilots for piloting qualitative analytic methods; and 2) embed their chosen theory, model or framework (TMF) throughout the qualitative study design and assess how themes or findings connect or interact across multiple domains. Researchers interested in these approaches can utilize the new library of PRISM-infused qualitative interview and focus group guides, of which the interview guides from this study are included: https://re-aim.org/prism-qualitative-database/.

### 4.2 Limitations

As noted, the primary interviewer is a developer of CDST. We aimed to reduce potential bias or impact on participants throughout the study (e.g., relationship building, explicitly asking for negative feedback) and received both positive and negative responses during interviews. However, this still may have influenced participant responses. Some aspects of our findings were likely impacted by the COVID-19 pandemic and associated restrictions; it may be that future work will lead to different reflections on telehealth as its capacity and comfort grows. Research is optional and some Veterans opted not to participate, usually citing lack of time or concerns about the privacy of their data. We minimized inclusion/exclusion criteria and only compensated for assessments to maximize representativeness, but future work will also assist with understanding potential representativeness or generalizability of these pilot findings within the population of Veterans who participate in PRRCs broadly.

## 5 Conclusion

Our strategy to embed PRISM throughout this qualitative study resulted in a nuanced understanding of initial feasibility and implementation determinants of CDST among Veterans with psychosis participating in VA PRRCs. We identified five cross-cutting themes which suggest that CDST is well-fitting for the recovery orientation and day-to-day structure of PRRCs, but that long-term sustainment is difficult for any intervention without infrastructural supports. Identifying mechanisms to gain and maintain these supports will be key for long-term success in the PRRC setting.

## Data Availability

The raw data supporting the conclusions of this article will be made available by the authors, without undue reservation.

## Supplementary material

SUPPLEMENTARY DATA SHEET 1 Interview Guides.

SUPPLEMENTARY DATA SHEET 2 Reporting Criteria.

SUPPLEMENTARY DATA SHEET 3 All Themes.
