# Supplementary material for: Feasibility of implementing collaborative decision skills training among veterans with psychosis: a qualitative study guided by the practical, robust implementation and sustainability model
Source: Front Health Serv. 2026 Aug 26;6:1837307. doi: 10.3389/frhs.2026.1837307 (PMC13553897; doi:10.3389/frhs.2026.1837307)
Supplement: Supplementary file 1 [file Datasheet1.docx]

*Interview Guide – Pre-Intervention (Baseline) Veteran Qualitative Assessment:*

Thank you for agreeing to talk with me today. I want to have a conversation with you about your mental health, and your experiences in mental health care. In particular, I’m interested in talking to you about how decisions related to your mental health are made right now, and whether you would like those decisions or the decision-making process to be different.

In our discussion today there are no wrong answers, and I would appreciate your honesty. Negative perceptions are just as welcomed as positive ones. Your individual responses will be kept confidential, and only used by research staff to understand perceptions of Veterans at CORE. I am going to audiotape the sessions so I can capture what you tell me. The interview should take about an hour.

Do you have any questions for me before we get started?

1. How would you describe your overall experience in mental health treatment? At CORE in particular? [Reach, Effectiveness]
   a. [prompt: How satisfied are you with these experiences? More or less satisfied at CORE compared to other services? How satisfied are you with how you have been treated?]
2. Ideally, how would you like the process of making a treatment decision to go? [Perspective of recipients, Characteristics of recipients]
   a. [prompt: How involved do you want to be? Who else do you want involved? Who should make the final decision? Do your answers depend on the decision being made?]
3. What is most important to you about the decision-making process? [Perspective of recipients, Characteristics of recipients]
   a. [prompt: options might include getting the treatment you most want, feeling heard or respected by your providers, or getting your provider’s expert recommendations- are any of those important to you? Why or why not?]
4. When you are making mental health decisions with your providers, how does that usually go? How close is the current process to your ideal process? [Perspective of recipients, Characteristics of recipients]
   a. [prompt: If your ideal treatment decision-making process doesn’t always occur, what gets in the way?]
5. How do providers act when treatment decisions are being made? [Perspective of recipients, Characteristics of recipients]
   a. [prompt: How does it depend on which provider you are working with? How does it change depending on the type of decision being made?]
6. How would being more involved in treatment decisions change your experience in mental health treatment? [Perspective of recipients]
   a. [prompt: Would it help you get your needs met? Help you achieve your treatment goals? Make you feel better in some way?]
7. What would need to change in order for you to be more involved in treatment decisions? [Perspective of recipients, Characteristics of recipients, Characteristics of implementers, Implementation & sustainability infrastructure]
   a. [prompt: Would you need to do something differently? Would your providers need to do something differently? Something else?]
8. Tell me a bit about your current engagement at CORE. [Characteristics of recipients]
   a. [prompt: Which treatment activities are you actively involved in now? Who provides them? How often do you attend? How were those activities chosen? What do you like or dislike about them?]
9. Do you ever have difficulty being engaged in services? What about difficulty attending appointments or doing out of appointment practice? What gets in the way of engaging? [Characteristics of recipients]
   a. [prompt: What would need to change in order for you to be more active with treatment services at CORE?] [Perspective of recipients, Characteristics of recipients, Characteristics of implementers, Implementation & sustainability infrastructure]

Any final thoughts?

Thank you very much for your time.

*Interview Guide – Post-Intervention Veteran Qualitative Assessment:*

Thank you for agreeing to talk with me today. I want to have a conversation with you about your experience during the study, including your perceptions of CDST, and any changes you’ve noticed in your mental health since the study began.

In our discussion today there are no wrong answers, and I would appreciate your honesty. Negative perceptions are just as welcomed as positive ones. Your individual responses will be kept confidential, and only used by research staff to understand perceptions of Veterans at CORE. I am going to audiotape the sessions so I can capture what you tell me. The interview should take about an hour.

Do you have any questions for me before we get started?

1. What changes have you noticed in yourself since we last talked about two months ago?   [Effectiveness, Perspective of recipients, Characteristics of recipients]
a. [prompt about changes in mental health, mental health treatment, and progress on treatment goals. Can prompt about why Veteran believes change occurred.]
2. Have any changes in your treatment been made?   [Effectiveness, Perspective of recipients]
a. Prompt: What were they? Why were those decisions made? Who initiated those changes/participated in those decisions? How satisfied are you with those decisions?
3. How are decisions made related to your mental health treatment?   [Effectiveness, Perspective of recipients,]
a. Prompt: Have there been any changes made to how you and your providers make treatment decisions?
b. [prompt about asking questions, making requests, stating preferences, and stating agreement/disagreement]
4. How satisfied are you with how treatment decisions are made? Are you more or less satisfied than you were when the study started?   [Effectiveness, Perspective of recipients]
5. In general, how would you describe your relationships with your providers now?   [Effectiveness, Perspective of recipients, Characteristics of recipients]
a. Prompt: Have you noticed any changes in how you approach interacting with your providers?
b. Prompt: How would you describe those changes? How have your providers responded to you?
6. Have you noticed any changes in your engagement in mental health care?   [Effectiveness, Perspective of recipients]
a. Prompt: At CORE specifically?
b. Prompt: What about changes in which activities you engage in? How often do you attend? How often do you complete out-of-appointment practice?
7. Have you tried to use any of the things you learned in CDST?  [Effectiveness]
a. [If yes: what did you do and how did it go? Prompt discussion of provider responsiveness.]
b. [If no: why not? Do you think you will try to use any of these things in the future?]
8. How was your experience in CDST?   [Perspective of recipients]
a. Prompt: How was your experience with your CDST therapist?
9. Do you think that CDST helped you?   [Effectiveness, Perspective of recipients]
a. [If yes: how?]
10. Do you think CDST negatively impacted you?   [Perspective of recipients, Effectiveness- adverse effects]
a. [If yes: how?]
11. What did you like about CDST?   [Perspective of recipients]
12. What didn’t you like about CDST?   [Perspective of recipients]
a. Prompt: What would have improved your experience in CDST?

Any final thoughts?

Thank you very much for your time.

*Interview Guide – 3 Month Follow-Up Veteran Qualitative Assessment:*

Thank you for agreeing to talk with me today. We met last about three months ago to discuss your experiences in our study, and particularly your experience in CDST. As a refresher, you participated in 9 group sessions of CDST led by [Vanessa or Sam], which focuses on learning skills to be more involved in treatment decision making. Today, I want to follow-up with you to hear about how you are doing, along with how things are going at CORE, and your thoughts about CDST now.

In our discussion today there are no wrong answers, and I would appreciate your honesty. Negative perceptions are just as welcomed as positive ones. Your individual responses will be kept confidential, and only used by research staff to understand perceptions of Veterans at CORE. I am going to audiotape the sessions so I can capture what you tell me. The interview should take about an hour.

Do you have any questions for me before we get started?

1. What changes have you noticed in yourself since we last talked about three months ago?   [Effectiveness, Perspective of recipients, Characteristics of recipients]
a. [prompt about changes in mental health, mental health treatment, and progress on treatment goals. Can prompt about why Veteran believes change occurred.]
2. Have any changes in your treatment been made?   [Effectiveness, Perspective of recipients]
a. Prompt: What were they? Why were those decisions made? Who initiated those changes/participated in those decisions? How satisfied are you with those decisions?
3. How are decisions made related to your mental health treatment?   [Effectiveness, Perspective of recipients]
a. Prompt: Have there been any changes made to how you and your providers make treatment decisions?
b. [prompt about asking questions, making requests, stating preferences, and stating agreement/disagreement]
4. How satisfied are you with how treatment decisions are made? Are you more or less satisfied than you were when the study started?   [Effectiveness, Perspective of recipients]
5. In general, how would you describe your relationships with your providers now?   [Effectiveness, Perspective of recipients, Characteristics of recipients]
a. Prompt: Have you noticed any changes in how you approach interacting with your providers?
b. Prompt: How would you describe those changes? How have your providers responded to you?
6. Have you noticed any changes in your engagement in mental health care?   [Effectiveness, Perspective of recipients]
a. Prompt: At CORE specifically?
b. Prompt What about changes in which activities you engage in? How often you attend? How often you complete out-of-appointment practice?
7. Have you tried to use any of the things you learned in CDST?  [Effectiveness]
a. [If yes: what did you do and how did it go? Prompt discussion of provider responsiveness.]
b. [If no: why not? Do you think you will try to use any of these things in the future?]
8. How was your experience in CDST?   [Perspective of recipients]
a. Prompt: How was your experience with your CDST therapist?
9. Do you think that CDST helped you?   [Effectiveness, Perspective of recipients]
a. [If yes: how?]
b. Prompt: Do you think CDST negatively impacted you?   [Effectiveness- adverse effects, Perspective of recipients]
c. [If yes: how?]
10. If CDST had been offered as a regular CORE group instead as a part of the study, would you be interested in participating? Would you recommend CDST to other Veterans?  [Perspective of recipients, Characteristics of recipients]
a. [If yes: What would you tell them about why they should participate in CDST?]  [Reach, Perspective of recipients, Characteristics of recipients]

Any final thoughts?

Thank you very much for your time.

*Interview Guide – Post-Intervention Feedback Qualitative Assessment (OPTIONAL / CDST GROUP ONLY)*

Thank you for agreeing to talk with me today. We’ve already met to discuss your experiences during the study. Today, I’d like to spend our meeting discussing your feedback on CDST. As you know, CDST is a group intervention that is intended to help Veterans like you gain knowledge and skills to help them be more involved in treatment decisions. I’m interested in your thoughts about CDST generally, and particularly any suggestions you have to improve it.

In our discussion today there are no wrong answers, and I would appreciate your honesty. Negative perceptions are just as welcomed as positive ones. Your individual responses will be kept confidential, and only used by research staff. I am going to audiotape the sessions so I can capture what you tell me. The interview should take an hour.

Do you have any questions for me before we get started?

1. Overall, what did you think of CDST?  [Perspective of recipients]
a. Prompt: What were your favorite things about CDST? What were your least favorite things?
2. If you could change one thing to CDST to make it more helpful for you and other Veterans, what would it be? Why did you choose that?   [Perspective of recipients]
a. Prompt: Is there anything else you would ideally want to change? Why?
3. If you could give CDST therapists one piece of advice, what would it be? Why?  [Perspective of recipients, Implementation, Effectiveness]
4. What parts of CDST were the most challenging to participate in? Why?  [Perspective of recipients, Implementation]
a. Prompt: Are there any changes we could to make it easier for you to participate in?
5. Do you think that CDST helps Veterans? How?   [Perspective of recipients, Effectiveness]
   a. Prompt: Which Veterans do you think are most likely to benefit from attending CDST?  [Characteristics of recipients]
6. Do you think that Veterans will be interested in participating in CDST? Why or why not? [Perspective of recipients, Reach]
a. Prompt: What might make Veterans less interested or less able to participate? Is there anything we should do to help Veterans participate? [Reach, Implementation]
7. Do you think that CDST should be offered regularly to Veterans at CORE? Why or why not?   [Implementation, Perspective of recipients]
8. What did you think of the at-home practice assignments?  [Perspectives of recipients, Effectiveness]
a. Prompt: Any you really liked or didn’t like? Any you struggled to complete?
b. Prompt: If you struggled to complete at home practice, why do you think that was? Is there anything that would help you get at home practice done more frequently?
c. Prompt: Did you ever attend Practice Group to help with getting at home practice done? Was that helpful? What could we do to make Practice Group more helpful?
9. What did you think of the skills taught during CDST, including NOW, ASAP, and SCALIE?  [Perspectives of recipients, Effectiveness]
10. What did you think of the role-plays and other activities you did during group?  [Perspectives of recipients, Effectiveness]
11. What did you think that the examples and stories in the group? [Perspectives of recipients, Effectiveness]
a. Prompt: Were they applicable to you? Were there any that really didn’t work for you?
12. What did you think of the CDST Handbook, the CDM Basics Card, and the Coping Skills Card?   [Perspectives of recipients, Effectiveness]
13. What did you think of CDST’s structure, including the number of sessions and length of sessions?  [Perspectives of recipients, Implementation]

Any final thoughts?

Thank you very much for your time.

*Interview Guide: Pre-Intervention Clinician Qualitative Assessment*

Thank you for agreeing to talk with me today. Today, I’d like to spend our meeting discussing your thoughts on CDST and how it might work for Veterans at CORE and fit into the CORE program. As a reminder, CDST is Collaborative Decision Skills Training, a 10-session skills training group that focuses on improving Veteran’s knowledge, skills, comfort, and confidence related to treatment decision-making. CDST’s core components include identification of treatment goals and treatment team members, assertiveness and communication skills, navigating conflict and disagreements, and problem-solving, all within an empowerment and recovery-oriented perspective. CDST will be delivered weekly by you or another CORE clinician, virtually during the pandemic, or in person afterwards. Veterans will be assigned at home practice for the first seven sessions. Veterans will be offered a drop-in 30 minute practice group after each session to work on their at home practice from CDST or other groups.

In our discussion today there are no wrong answers, and I would appreciate your honesty. Negative perceptions are just as welcomed as positive ones. Your individual responses will be kept confidential, and only used by research staff. I am going to audiotape the sessions so I can capture what you tell me. The interview should take about 30 minutes. Do you have any questions for me before we get started?

1. Overall, how do you anticipate delivering CDST to CORE Veterans will go?
a. Prompt: What might go well? What might go poorly?
2. Which CORE Veterans will benefit more than others from CDST? [RE-AIM Effectiveness – equitable benefit]
a. Prompt: What benefits might Veterans gain from CDST?
3. How do you think participating in CDST might negatively impact the Veterans in your group? [RE-AIM Effectiveness – Adverse outcomes]
4. What elements of CDST might be most challenging to deliver? Why? [PRISM context – Intervention from perspective of implementer]
5. What elements of CDST do you anticipate Veterans will have the toughest time engaging in? Why? [PRISM context –Intervention from perspective of implementer]
6. What are the most likely roadblocks you think we might face when delivering CDST to CORE Veterans?   [Implementation and sustainability infrastructure]
a. Prompt: Are these roadblocks specific to CDST, or are they something you face with most CORE groups?
b. Prompt: Are these roadblocks solvable? How?
7. How well does the proposed delivery of the CDST fit with the current workflow at the CORE? [PRISM context – Intervention characteristics – perspective of implementor AND Implementation and sustainability infrastructure]
8. *How well does the CDST delivery align with existing resources at the CORE? [PRISM context – Intervention characteristics – perspective of implementer AND Implementation and sustainability infrastructure]
a. What resources might CDST require that CORE doesn’t already have?
9. *What are some key challenges you anticipate with the ongoing/sustained delivery of CDST at the CORE? [RE-AIM - Sustainment/maintenance]
a. Do you think that CDST will be a treatment that will eventually be offered regularly to Veterans at CORE? What would keep CDST from becoming a part of the regular offerings?

Any final thoughts?

Thank you very much for your time.

*Interview Guide: Post-Intervention Clinician Qualitative Assessment*

Thank you for agreeing to talk with me today. Today, I’d like to spend our meeting discussing your feedback on CDST. As you know, CDST is a group intervention that is intended to help Veterans at CORE gain the knowledge and skills to be more involved in treatment decisions. I’m interested in your thoughts about CDST generally, and particularly, any suggestions you have to improve it.

In our discussion today there are no wrong answers, and I would appreciate your honesty. Negative perceptions are just as welcomed as positive ones. Your individual responses will be kept confidential, and only used by research staff. I am going to audio record the sessions so I can capture what you tell me. The interview should take about an hour.

Do you have any questions for me before we get started?

1. Overall, how did the delivery of CDST go (at CORE)?***
a. Prompt: What went well? What went poorly?/What was challenging? Do you have a sense about why some things went better than others?
2. Which Veterans do you think benefited most from CDST? Why?
a. Prompt: What benefits did they gain? What did you notice about them? Based on this, are there particular types of Veterans you might recommend or not recommend for CDST?
b. [prompt regarding decision-making approach, assertiveness, treatment engagement, progress on goals]

[RE-AIM Effectiveness – equitable benefit]

3. Which elements of CDST seemed to have the most positive impact on Veterans?***
a. Prompt: What impacts did you notice?

[PRISM context – Intervention characteristics – Veteran’s perspective]

4. Did any Veterans experience a negative impact due to participating in CDST?***
a. Prompt: What were these negative impacts?
b. Prompt: Overall, are you concerned about adverse outcomes due to participation in CDST? Are there particular Veterans who you might be more concerned about adverse outcomes?

[RE-AIM Effectiveness – Adverse outcomes]

5. Which elements from CDST were most challenging to deliver? What made them challenging?***
a. Prompt: Are there any changes that could be made to increase ease of delivery?

[PRISM context – Intervention from perspective of implementer AND Implementation]

6. Which elements of CDST did Veterans have the toughest time engaging in? Why do you think they struggled with it?
a. Prompt: Are there any changes that could be made to help Veterans engage in these elements more easily?

[PRISM context – Intervention characteristics – Veteran’s perspective]

7. Overall, what elements of CDST would you recommend modifying?***
a. Prompt: Think about the content in the manuals, the at home practice assignments, the number of sessions and length of sessions
b. Why do you suggest modifications to these elements?

[PRISM context – Intervention from perspective of implementer]

8. Before you delivered CDST, you said you thought that _________ would be the most likely roadblock we might face when delivering CDST to CORE Veterans. Did you experience this?
a. If yes: Why? Was it due to the same causes you imagined? Is the roadblock solvable? How?
b. If no: Why do you think it didn’t occur? Do you think you would have experienced this roadblock in another CORE group [is this success specific to CDST]? How can we replicate this success?

[PRISM: relevant constructs they mentioned will define what PRISM domains are impacted, will typically include implementation & sustainability infrastructure; effectiveness or implementation]

9. What response, if any, did you notice from other clinicians at CORE to CDST? What about responses to Veterans attempting collaborative decision-making?
a. Prompt:  What role might clinicians have in CDST’s success, both in terms of it continuing at CORE and in terms of it benefiting Veterans?  Are there ways to improve that role?

[PRISM context – Intervention characteristics – perspective of implementer AND Implementation and sustainability infrastructure]

10. Did the delivery of the CDST fit with the current workflow at the CORE?
a. Prompt: Are there any changes you recommend to improve fit?

[PRISM context – Intervention characteristics – perspective of implementer AND Implementation and sustainability infrastructure]

11. How well does the CDST delivery align with existing resources at the CORE?
a. Prompt: What resources does CDST require that CORE doesn’t already have?

[PRISM context – Intervention characteristics – perspective of implementer AND Implementation and sustainability infrastructure]

12. What are some key challenges you anticipate with the ongoing/sustained delivery of CDST at the CORE?***
a. Prompt: Do you think that CDST will be a treatment that will eventually be offered regularly to Veterans at CORE? What would keep CDST from becoming a part of the regular offerings?
b. Prompt: We’ve talked about tangible resources, but what about less tangible things that might get in the way?

[RE-AIM - Sustainment/maintenance] and PRISM Implementation and sustainability infrastructure]

Any final thoughts?

Thank you very much for your time.

*Interview Guide: Baseline Clinician Qualitative Assessment - FOR CDST EXPERIENCED CLINICIANS*

Thank you for agreeing to talk with me today. Today, I’d like to spend our meeting discussing your thoughts on CDST and how it might work for Veterans at CORE and fit into the CORE program based on your past experience with CDST and the changes we’ve reviewed. As a reminder, CDST is Collaborative Decision Skills Training, an 10-session skills training group that focuses on improving Veteran’s knowledge, skills, comfort, and confidence related to treatment decision-making. CDST’s core components include identification of treatment goals and treatment team members, assertiveness and communication skills, navigating conflict and disagreements, and problem-solving, all within an empowerment and recovery-oriented perspective. For this round, CDST will be delivered weekly by you [virtually/in person]. Veterans will be assigned at home practice for the first nine sessions.

In our discussion today there are no wrong answers, and I would appreciate your honesty. Negative perceptions are just as welcomed as positive ones. Your individual responses will be kept confidential, and only used by research staff. I am going to audiotape the sessions so I can capture what you tell me. The interview should take about 30 minutes.

Do you have any questions for me before we get started?

1. Overall, how do you anticipate delivering CDST to CORE Veterans will go?
a. Prompt: What might go well? What might go poorly?
2. Based on the changes we made to CDST since the last time you delivered, how do you think delivering CDST might change?
a. Prompt: What might be easier to deliver? What might be harder?
b. Prompt: What might be more effective? What might be less effective?
3. Which CORE Veterans will benefit more than others from CDST? *[RE-AIM Effectiveness – equitable benefit]*
a. Prompt: What benefits might Veterans gain from CDST?
b. Prompt: Based on your past experience, what clinical approach do you plan to use to maximize benefits to Veterans? Is this approach the same or different than what you did in the past? How well does it align with the CDST model?
4. How do you think participating in CDST might negatively impact the Veterans in your group? *[RE-AIM Effectiveness – Adverse outcomes]*
a. Prompt: : Based on your past experience, what clinical approach do you plan to use to identify negatively impacted Veterans and assist them? Is this approach the same or different than what you did in the past? How well does it align with the CDST model?
5. What elements of CDST might be most challenging to deliver? Why? *[PRISM context – Intervention from perspective of implementer]*
a. Prompt: Based on your past experience, what clinical approach do you plan to use to increase ease of delivery? Is this approach the same or different than what you did in the past? How well does it align with the CDST model?
6. What elements of CDST do you anticipate Veterans will have the toughest time engaging in? Why? *[PRISM context – Intervention characteristics – Veteran’s perspective]*
a. Prompt: Based on your past experience, what clinical approach do you plan to use to increase ease of engagement for Veterans? Is this approach the same or different than what you did in the past? How well does it align with the CDST model?
7. What are the most likely roadblocks you think we might face when delivering CDST to CORE Veterans?
a. Prompt: Do you expect the same roadblocks you faced during your last delivery of CDST?
i. If so, why? What solutions did you try previously? What resources do you need for a solution to be successful?
ii. If not, why not?
b. Prompt: Are these roadblocks specific to CDST, or are they something you face with most CORE groups?
c. Prompt: Are these roadblocks solvable? How?
8. How well does the proposed delivery of the CDST fit with the current workflow at the CORE? *[PRISM context – Intervention characteristics – perspective of implementor AND Implementation and sustainability infrastructure]*
9. *How well does the CDST delivery align with existing resources at the CORE? *[PRISM context – Intervention characteristics – perspective of implementer AND Implementation and sustainability infrastructure]*
a. What resources might CDST require that CORE doesn’t already have?
10. *What are some key challenges you anticipate with the ongoing/sustained delivery of CDST at the CORE? *[RE-AIM - Sustainment/maintenance]*
a. Do you think that CDST will be a treatment that will eventually be offered regularly to Veterans at CORE? What would keep CDST from becoming a part of the regular offerings?

Any final thoughts?

Thank you very much for your time.
