# Supplementary material for: Feasibility of implementing collaborative decision skills training among veterans with psychosis: a qualitative study guided by the practical, robust implementation and sustainability model
Source: Front Health Serv. 2026 Aug 26;6:1837307. doi: 10.3389/frhs.2026.1837307 (PMC13553897; doi:10.3389/frhs.2026.1837307)
Supplement: Supplementary file 3 [file Datasheet3.docx]

All Themes and Domains

Not italicized = Theme ranked 1-3, including in paper either on its own or within cross-cutting themes

*Italicized* = Theme ranked 4 or below, not included in paper

| Domain | Themes |
| --- | --- |
| PRISM Context – Implementation and Sustainability Infrastructure | - CDST content, structure, and delivery aligns with PRRC clinical delivery - CDST delivery should be required by CORE due to program fit and benefit to both Veterans and clinicians - Practice group requires adjustment to fit sustainably into PRRC context - *Successful long-term implementation can be ensured by making CDST a PRRC requirement* |
| PRSIM Context – Intervention Characteristics – Perspective of Implementer | - Clinician anxiety and lack of training in experiential therapies are barriers to delivery of CDST role-plays - CDST is aligned with recovery model - Re-distribution of therapy components across first few sessions could decrease overstuffed sessions and improve ease of delivery - *CDST offers the most engagement to people in the VA/CORE setting* - *Repetition of coping skills throughout CORE programing as a barrier to participant engagement in such skills* - *Homework completion is a broad, ongoing issue but a practice group may be the solution to this* - *Veterans require additional assistance to help them understand and feel comfortable engaging in CDST role-plays* - *Clinician level of interest influences whether and how well an intervention is delivered* |
| PRISM Context – Intervention Characteristics – Veteran’s Perspective | - At-home practice was relevant and reasonable, but individualized barriers including scheduling overwhelm and difficulty remembering often got in the way - Veterans responded positively to many of the specific elements of the intervention - Virtual groups and smaller groups could present a barrier to engagement, especially with vignettes and roleplays - *Accountability is seen as an important and desired attribute in CDST delivery* - *Mixed review on practice group, thought it would be helpful but some may not need to utilize it* - *Some Veterans had difficulty with the treatment team part of CDST because they weren’t sure about which providers were on their teams* - *Reminder calls are helpful tools for addressing scheduling barriers and increasing CDST attendance* - *In person sessions are preferred and may improve group dynamics.* |
| PRISM Recipients – Organizational Characteristics | - Group offerings rely on both clinician and Veteran interest - Making CDST a required CORE group to ensure sustainability - Using increased structure to support maintenance of CDST given its similarity to existing CORE groups - *Feedback as a determinant for CDST implementation at CORE* - *Creating clear connections between CDST and relevant outcomes for VA clinicians and administrators is a key implementation determinant* - *CDST would fit well as an introductory group to CORE* |
| PRISM Recipients – Patient Characteristics | - Military culture, personal beliefs, and other past experiences could serve as barriers - Logistical factors including scheduling impact engagement - In person versus virtual delivery - *Some Veterans may speak up only when they disagree with their clinician* - *Core beliefs as barriers to CDST effectiveness* - *No one should be counted out of CDST* |
| RE-AIM Reach | - CDST may be helpful for those wanting to take charge in recovery - Veterans may not seek out CDST because it is a group setting - Word of mouth as a way to expand reach - *Unsolvable limits on reach* |
| RE-AIM Effectiveness | - Not included in this paper |
| RE-AIM Effectiveness – Adverse Outcomes | - Not included in this paper |
| RE-AIM Effectiveness – Equitable Benefit | - Not included in this paper |
| RE-AIM Adoption | - Clinicians may prefer to lead shorter groups - Logistics and interest are key to adoption - Practice group is novel and promising - *CDST has similar features to other CORE groups and fits in with other group offerings* |
| RE-AIM Implementation | - Mixed opinions about group structure (e.g. session length, number of sessions) and size - Poor at home practice completion - Mixed perceptions on the virtual delivery of CDST - *Veteran perceptions of clinicians impact overall group quality* - *Group dynamics and engagement* - *Integration of coping skills in each CDST session as a structural benefit* - *Changing the structure of CDST practice groups to increase Veteran benefit* - *Clinician ease of delivery to provide individually tailored support* - *There should be more sessions because CDST went too fast* |
| RE-AIM Sustainment/Maintenance | - Veteran and clinician interest as a major determining factor - Clarifying CDST’s role in Veteran recovery to enhance sustained delivery - Phase system may increase sustainability - *Structural factors supporting sustainment* - *Veterans think CDST should be offered regularly* |
